# Editorial: Role of Neuroinflammation in the Neuropsychiatric and Neurological Aspects of COVID-19

**DOI:** 10.3389/fncel.2022.840121

**Published:** 2022-02-09

**Authors:** Marie-Eve Tremblay, Charlotte Madore, Li Tian, Alexei Verkhratsky

**Affiliations:** ^1^Axe Neurosciences, Centre de Recherche du CHU de Québec, Université Laval, Québec City, QC, Canada; ^2^Neurology and Neurosurgery Department, McGill University, Montréal, QC, Canada; ^3^Department of Molecular Medicine, Université Laval, Québec City, QC, Canada; ^4^Division of Medical Sciences, University of Victoria, Victoria, BC, Canada; ^5^Department of Biochemistry and Molecular Biology, The University of British Columbia, Vancouver, BC, Canada; ^6^Univ. Bordeaux, INRAE, Bordeaux INP, NutriNeuro, UMR 1286, Bordeaux, France; ^7^Department of Physiology, Faculty of Medicine, Institute of Biomedicine and Translational Medicine, University of Tartu, Tartu, Estonia; ^8^Psychiatry Research Centre, Beijing Huilongguan Hospital, Peking University Health Science Center, Beijing, China; ^9^Faculty of Biology, Medicine and Health, The University of Manchester, Manchester, United Kingdom; ^10^Achucarro Center for Neuroscience, IKERBASQUE, Basque Foundation for Science, Bilbao, Spain; ^11^Department of Neurosciences, University of the Basque Country UPV/EHU and CIBERNED, Leioa, Spain; ^12^Sechenov First Moscow State Medical University, Moscow, Russia

**Keywords:** COVID-19, SARS-CoV-2, astrocytes, microglia, oligodendrocytes, comorbidity, development, aging

Neuroglial cells form homeostatic and defensive arms of the nervous system. In the central nervous system (CNS) neuroglia embrace astroglia, microglia, and cells of the oligodendroglial lineage, which all perform numerous vital functions in the healthy and diseased brain. Astroglia (which comprise various types of parenchymal astrocytes, radial astrocytes, radial stem astrocytes, and ependymocytes) are integral partners of neural networks that control homeostasis of the CNS across molecular, cellular and network, metabolic, as well as whole brain levels of organization. Oligodendroglia, represented by oligodendroglial precursor cells (also known as NG2 glia) and mature oligodendrocytes support myelination critical for the CNS connectome. Mainly known for their involvement with axonal myelination during development and lifelong plasticity, the cells of the oligodendrocytic lineage are also emerging as key contributors to brain inflammation aimed to resolve various types of challenges. Microglia, the resident CNS immune cells, play many roles in the development, maturation, function, and plasticity of the brain across life.

This Research Topic focuses on the implication of CNS inflammatory responses mediated by microglia and astrocytes, in the neuropsychiatric and neurological aspects of COVID-19. Neuroglial involvement comprises: (i) concerted actions in coordination with cells of the oligodendrocytic lineage and the neurovascular unit, (ii) reactivity when challenged by viral infection with COVID-19, resulting in CNS inflammation and changes in brain circuits associated with neuropsychiatric or neurological disorders, (iii) outcomes when the nervous tissue challenged with COVID-19 infection further has to deal with psychological stress, pollution, dietary imbalance, trauma, injury, and aging, and (iv) consequences of social isolation and psychological stress, in combination COVID-19 infection.

The knowledge into the COVID-19-associated changes in non-neuronal cells is expected to contribute to the development of better-targeted therapies to preserve, improve or restore learning and memory, cognitive flexibility, stress resilience, sleep, metabolic fitness, and adaptation, among other essential CNS functions. Dedicated to this important research avenue, our Research Topic comprises six articles, four Review articles, one Opinion article, and one original Research Article, which are introduced in this Editorial.

Tremblay et al. overview the neurotropism of SARS-CoV-2, the implication of CNS inflammation, adaptive and innate immunity, autoimmunity, as well as astrocytic and microglial immune and homeostatic functions in the neurological and psychiatric aspects of COVID-19. In addition, consequences of SARS-CoV-2 infection arising during aging, in the presence of systemic comorbidities, and for the exposed pregnant mother and fetus are discussed.

Specifically focusing on microglia, Gonçalves de Andrade et al. review their roles in the neuroprotective and neurotoxic responses against CNS insults instigated by COVID-19. The authors also discuss how these responses may explain, at least partially, the neurological and psychiatric manifestations reported in patients with COVID-19 and the general population, including aged individuals and people with pre-existing conditions.

Delving further into the outcomes of COVID-19 and aging on microglia, Awogbindin et al. provide complementary insight into the mechanisms by which microglia could contribute to the pathophysiology of post-COVID-19 neurological sequelae and disorders, including the late-onset form of Parkinson's disease, which is expected to be pervasive in the coming years given the growing numbers of infected and re-infected individuals globally.

Shifting the focus to astrocytes, Tavčar et al. review the involvement of astrocytes in COVID-19 pathology. The present knowledge on SARS-CoV-2 as a neurotropic virus and on several other neurotropic flaviviruses is covered to highlight the neurotropic mechanisms targeting astroglial cells. The involvement of astrocytes, related to aging and neurodegenerative disorders, and environmental factors, is further discussed in the context of COVID-19.

Valenza et al. provide an opinion regarding the implication of systemic inflammation and astrocytic reactivity in the neuropsychiatric consequences of COVID-19. Deleterious outcomes of astrocytic reactivity during fetal development are particularly discussed, together with the risk of developing autism spectrum disorders in the infected progeny.

Finally, Yan et al. present data revealing an increased expression of receptors for SARS-CoV-2 in the prefrontal cortex of male, but not female mice exposed to chronic unpredictable stress. Similar sex-specific changes in SARS-CoV-2 receptors were also observed in monocytes from human caregivers enduring chronic stress. These findings indicate a potential sex-dependent susceptibility to SARS-CoV-2 infection after chronic stress.

## Author Contributions

M-ET and AV wrote the manuscript, which was revised by all the authors. All authors contributed to the article and approved the submitted version.

## Conflict of Interest

The authors declare that the research was conducted in the absence of any commercial or financial relationships that could be construed as a potential conflict of interest.

## Publisher's Note

All claims expressed in this article are solely those of the authors and do not necessarily represent those of their affiliated organizations, or those of the publisher, the editors and the reviewers. Any product that may be evaluated in this article, or claim that may be made by its manufacturer, is not guaranteed or endorsed by the publisher.

